# S6K1 Is Indispensible for Stress-Induced Microtubule Acetylation and Autophagic Flux

**DOI:** 10.3390/cells10040929

**Published:** 2021-04-17

**Authors:** Aleksandra Hać, Karolina Pierzynowska, Anna Herman-Antosiewicz

**Affiliations:** 1 Department of Medical Biology and Genetics, Faculty of Biology, University of Gdańsk, Wita Stwosza 59, 80-308 Gdańsk, Poland; anna.herman-antosiewicz@ug.edu.pl; 2 Department of Molecular Biology, Faculty of Biology, University of Gdańsk, Wita Stwosza 59, 80-308 Gdańsk, Poland; karolina.pierzynowska@ug.edu.pl

**Keywords:** S6 kinase 1 (S6K1), tubulin acetylation, autophagic flux, autophagosome-lysosome fusion, serum deprivation, sulforaphane, lysosome

## Abstract

Autophagy is a specific macromolecule and organelle degradation process. The target macromolecule or organelle is first enclosed in an autophagosome, and then delivered along acetylated microtubules to the lysosome. Autophagy is triggered by stress and largely contributes to cell survival. We have previously shown that S6K1 kinase is essential for autophagic flux under stress conditions. Here, we aimed to elucidate the underlying mechanism of S6K1 involvement in autophagy. We stimulated autophagy in S6K1/2 double-knockout mouse embryonic fibroblasts by exposing them to different stress conditions. Transient gene overexpression or silencing, immunoblotting, immunofluorescence, flow cytometry, and ratiometric fluorescence analyses revealed that the perturbation of autophagic flux in S6K1-deficient cells did not stem from impaired lysosomal function. Instead, the absence of S6K1 abolished stress-induced tubulin acetylation and disrupted the acetylated microtubule network, in turn impairing the autophagosome-lysosome fusion. S6K1 overexpression restored tubulin acetylation and autophagic flux in stressed S6K1/2-deficient cells. Similar effect of S6K1 status was observed in prostate cancer cells. Furthermore, overexpression of an acetylation-mimicking, but not acetylation-resistant, tubulin variant effectively restored autophagic flux in stressed S6K1/2-deficient cells. Collectively, S6K1 controls tubulin acetylation, hence contributing to the autophagic flux induced by different stress conditions and in different cells.

## 1. Introduction

The cell experiences different stresses during its lifetime. Under adverse conditions, it activates a number of processes to allow its adaptation and ensure survival. One such process is autophagy (literally, “self-eating”), i.e., lysosomal degradation of macromolecules and even whole organelles. Macroautophagy (hereafter referred to as “autophagy”) is essential for maintaining basal cellular homeostasis, by enabling macromolecule recycling. This type of autophagy constantly takes place in a cell under non-stress conditions and is called “basal” autophagy [1]. However, numerous stress stimuli enhance the autophagy rate, resulting in “induced” autophagy [1]. Induced autophagy is crucial for sustaining cell homeostasis during stress conditions and eventually cell survival, as it enables the clearance of pathogens, abnormal and aggregated proteins, or damaged organelles that are potentially harmful to a cell (for a review, see [2]). Even though autophagy is predominantly a pro-survival process, excessive autophagy rate or duration can eventually lead to programmed cell death, hence it might serve as a strategy to induce death of cancer cells with defective apoptosis process. Defects in autophagy are observed in many pathological conditions, including neurodegenerative diseases, myopathies, and cancer (for a review, see [3]). Both, the induction and inhibition of autophagy are of great therapeutic importance, but their regulation on the molecular level has not yet been fully elucidated.

Autophagy entails several sequential steps: sequestration, fusion, degradation, and macromolecule recycling. First, a portion of the cytoplasm is engulfed by a membrane fold (called the isolation membrane) and enclosed in a double-membrane autophagosome. Next, it is transported along acetylated microtubules to a lysosome, with which it fuses, forming an autolysosome. Lysosomal hydrolases digest both autophagosome contents and its inner membrane, and the released molecules are recycled and reused. The equilibrium between autophagosome formation and clearance by lysosomes is termed the “autophagic flux” [4,5,6]. 

Acetylated microtubules are essential for the autophagosome transport towards a lysosome and their fusion [7]. Acetylation of Lys40 of α-tubulin alters microtubule properties, and is controlled by the balance between the activities of αTAT1 (α-tubulin N-acetyltransferase 1; also termed ATAT1 or MEC17) and deacetylases HDAC6 (histone deacetylase 6) and Sirt2 (NAD-dependent deacetylase sirtuin 2) [8,9]. Although microtubules dynamically switch between growing and shrinking phases, certain microtubules can be preferentially stabilized by post-translational modifications or interactions with proteins. Tubulin acetylation occurs on such stable microtubules, which protects them against microtubule-depolymerizing drugs; however, the exact mechanism of this phenomenon is not clear. It is speculated that tubulin acetylation affects a set of proteins associated with the cytoskeleton or microtubule conformation [10,11,12,13]. 

Enhanced microtubule acetylation (hyperacetylation) is a rapid and general cell response to various stressors and increases cell survival [7,12,14]. It enables efficient binding of dynein, a microtubular motor protein, which is crucial for autophagosome transport toward the lysosome under stress conditions [14,15]. Among the functional consequences of the tubulin acetylation status are alterations in microtubule dynamics and stability, cell migration, and autophagy [12,16,17]. 

Numerous signaling pathways are involved in the control of autophagy. Most of them converge at the mammalian/mechanistic target of rapamycin complex 1 (mTORC1), the main autophagy regulator (for a review, see [18]). mTOR is an evolutionarily conserved protein kinase that integrates signals on the availability of nutrients, hormones, and growth factors. When active, mTORC1 promotes cell growth and proliferation, and simultaneously inhibits autophagy [19]. One of the best-studied mTORC1 substrates is ribosomal S6 protein kinase 1 (S6K1), a serine/threonine kinase activated in response to nutrients and growth factors. S6K1 stimulates protein synthesis and cell growth by, among other, phosphorylating the ribosomal S6 protein [20]. Analysis of S6K1^−/−^ knockout model led to the discovery of S6K2, an S6K1 homolog. In mammalian cells, decreased S6K1 expression is often compensated by S6K2 overactivation [20]. However, despite a high degree of homology, S6K1 and S6K2 share only some substrates and functions [21]. A link between the overexpression of S6K1 and S6K2 kinases and breast or prostate cancers has been identified. Further, S6K1/2 overexpression is correlated with a poor response to treatment and overall prognosis [22,23,24].

Whereas the role of mTORC1 in autophagy is well established, the role of S6K1 in this process is elusive and controversial. For instance, studies involving rat hepatocytes indicate that active S6K1 suppresses autophagy, as shown by an inverse linear correlation between the degree of phosphorylation of an S6K1 substrate, the ribosomal S6 protein, and autophagic flux [25,26]. Furthermore, in *Drosophila* and mammalian cells, ATG1 (Ulk1), a protein essential for the induction of autophagy, specifically inhibits S6K1 activity by blocking its phosphorylation at Thr389 [27]. By contrast, Scott and collaborators proposed that S6K1 promotes rather than suppresses autophagy, as lack of S6K1 limits autophagy during extended starvation in the fat body of *Drosophila* [28] and disrupts the process in mammalian cells [29]. Of note, the observation that downregulation of S6K1 leads to S6K2 overactivation in mammalian cells [20] additionally complicates the issue and data interpretation. The impact of S6K2 on autophagy has not been investigated in detail.

Previously, using prostate cancer cells (PC3) with silenced S6K1 expression and S6K1/2 double-knockout mouse embryonic fibroblasts (S6K DKO MEF), we showed that in cells devoid of S6K1, while the efficiency of lysosomal degradation of the autophagosome (autophagic flux) is not perturbed under basal conditions, it is impaired under stress conditions induced by serum deprivation or by sulforaphane (SFN) [30]. The latter is a natural autophagy-inducing agent in cancer and non-cancerous cells [30,31]. Overexpression of exogenous S6K1 in S6K DKO MEF restored the autophagic flux, but the effect of S6K2 reintroduction was insignificant [30]. These observations indicate that S6K1, but not S6K2, plays a role in autophagosome maturation but only under stress conditions. However, the questions regarding S6K1 indispensability for autophagy only under stress and the underlying mechanism by which S6K1 affects the final stages of autophagy remain unanswered. Therefore, the present study was designed to investigate the mechanism underpinning the observed impaired autophagosome maturation in S6K1/2-deficient cells under stress conditions induced by various cell stressors, including SFN, rapamycin, and either glucose or growth factors deprivation. We show that the perturbation of autophagic flux in S6K1-deficient cells did not stem from impaired lysosomal function. Instead, the absence of S6K1 abolished stress-induced tubulin acetylation and disrupted the acetylated microtubule network, in turn impairing the autophagosome-lysosome fusion. This effect was observed in autophagy induced by different stress conditions and in two different cell models which strongly suggests that it is a general phenomenon. To the best of our knowledge, this is the first report on the impact of S6K1 on tubulin acetylation and microtubular cytoskeleton network. As deregulation of S6K1 is observed in many disorders, including cancer, the findings of the current study might shed new light on the role of S6K1 in the pathomechanisms of such disorders.

## 2. Materials and Methods

### 2.1. Reagents

d,l-SFN (99% purity) was purchased from LKT Laboratories (St. Paul, MN, USA). Antibodies against p-S6K1 (Thr389; #sc-11759-R), green fluorescent protein (GFP; #sc-9996), and hemagglutinin (HA)-tag (#sc-7392), as well as control (#sc-37007) and S6K1-targeting siRNAs (#sc-36-165; a mixture of three different target-specific siRNAs), were form Santa Cruz Biotechnology (Dallas, TX, USA). Antibodies against S6K1 (#04-391) and p-S6 (Ser-240/244; #07-2113) were from Merck Millipore (Burlington, MA, USA). Antibodies against S6 (#2317), Beclin 1 (#3495), Ulk1 (#8054) and rapamycin were from Cell Signaling Technology (Danvers, MA, USA). Anti-LC-3 antibodies (#PM036) were from MBL International (Woburn, MA, USA), anti-ATG5 antibodies (#GTX113309)—from GeneTex (Irvin, CA, USA), and anti-LAMP1 (#ab24170)—from Abcam (Cambridge, UK). Antibodies against acetylated α-tubulin (#T7451; Clone 6-11B-1), α-tubulin (#T6199; clone DM1A), β-actin (#A3854), and GAPDH (#G9295); rabbit anti-mouse (#A9044) and goat anti-rabbit (#A9169) secondary antibodies conjugated to horseradish peroxidase; goat anti-mouse secondary antibodies conjugated to TRITC (#T5393); and dimethyl sulfoxide (DMSO) and chloroquine were from Sigma-Aldrich (Saint Louis, MO, USA). Plasmids encoding HA-tagged S6K1 [#8984, pRK7-HA-S6K1 WT; #8991, pRK7-HA-S6K1-F5A-E389-R3A (HA-S6K1 CA)] were constructed by Dr. John Blenis’s group [32] and obtained from Addgene (Watertown, MA, USA). Plasmids encoding GFP-tagged α-tubulin variants were a gift from Dr. Tso-Pang Yao and obtained from Addgene: pcDNA3.1(+)-EGFP-Tubulin.K40Q, encoding acetylation-mimicking variant (#32912), and pcDNA3.1(+)-EGFP-Tubulin.K40R, encoding acetylation-resistant variant (#30488) [33]. A plasmid encoding tandem-fluorescent LC3 (pBABE-puro-mCherry-EGFP-LC3B) was a gift from Dr. Jayanta Debnath (#22418) [34]. LysoSensor Yellow/Blue was from Thermo Scientific (Waltham, MA, USA). Glucose- and sodium pyruvate-free Dulbecco’s Modified Eagle Medium was from Biological Industries (Kibbutz Beit Haemek, Israel).

### 2.2. Cell Lines and Cell Culture

Monolayer cultures of mouse embryonic fibroblasts derived from WT or S6K1^−/−^ S6K2^−/−^ mice (WT MEF and S6K DKO MEF, respectively) [21] were kindly provided by Dr. Mario Pende. Cells were cultured in Dulbecco’s Modified Eagle Medium (high glucose, pyruvate-free; Sigma-Aldrich, Saint Louis, MO, USA) containing 10% (*v*/*v*) fetal bovine serum (FBS; Thermo Scientific, Waltham, MA, USA) and penicillin–streptomycin mixture (Sigma-Aldrich, Saint Louis, MO, USA). PC3 cells were kindly provided by Prof. Danuta Duś and were maintained in F12-K Nutrient Mixture medium (Thermo Scientific, Waltham, MA, USA) supplemented with 9% (*v*/*v*) FBS and penicillin-streptomycin antibiotics mixture. Each cell line was maintained at 37 °C in a humidified atmosphere with 5% CO_2._

MEF cell lines stably expressing tandem-fluorescent LC3 (mCherry-GFP-LC3) were generated by transfection with pBabe-puro-mCherry-EGFP-LC3B plasmid using TurboFect (Thermo Scientific, Waltham, MA, USA) and puromycin selection. After selection, stable cell lines were cultured in the medium *s*upplemented with 8 µg/mL and 4 µg/mL of puromycin (WT MEF and S6K DKO MEF cells, respectively). d,l-SFN was prepared in DMSO and stored as a 10–40 mM stock, at −20 °C. Chloroquine was prepared in dH_2_O immediately before use, as a 10 mM stock.

### 2.3. Transient Transfection with Plasmid DNA or siRNA

WT and S6K DKO MEF were transfected with an empty vector or a vector coding for HA-tagged WT S6K1 or GFP-tagged α-tubulin (acetylation-mimicking or acetylation-resistant variants) using TurboFect (Thermo Scientific, Waltham, MA, USA), according to the manufacturer’s instructions. PC3 cells were transfected with 50 nM control or S6K1-targeting siRNA (Santa Cruz Biotechnology, Dallas, TX, USA; sc-37007 and sc-36165, respectively) using LipoFectamine RNAiMax (Thermo Scientific, Waltham, MA, USA), according to the manufacturer’s recommendations. One to two days after plasmid transfection, or 72 h after siRNA transfection, the cells were treated with an autophagy inducer, as described in Section 2.4.

### 2.4. Stress Conditions for the Induction of Autophagy

For growth factor deprivation or glucose starvation, cells were washed with phosphate-buffered saline (PBS) and cultured in a medium without FBS for the indicated times (growth factors deprivation), or for 3 h in a medium without glucose, sodium pyruvate, and FBS (glucose starvation). Control cells were treated identically, except that a medium containing FBS or glucose (accordingly) was used. For SFN treatment, MEF or PC3 cells were exposed to 20 μM or 40 μM SFN, respectively, for the indicated times. Rapamycin was used at a concentration of 100 nM for 3 h. Control cells were treated with an equal volume of DMSO. To measure the autophagic flux, 10 µM chloroquine was added for the last 2 h of treatment, and the difference in the LC3-II levels in samples with or without the inhibitor (a measure of autophagic flux efficiency) were determined by immunoblotting (Section 2.5).

### 2.5. Immunoblotting

Cells treated with SFN or growth factor-deprived cells were lysed in a solution containing 50 mM Tris, 1% Triton X-100, 150 mM NaCl, 0.5 mM EDTA, and protease and phosphatase inhibitor cocktails (Roche Diagnostics, Basel, Switzerland). The lysates were cleared by centrifugation at 15,500× *g* for 20 min. Proteins were resolved by sodium dodecyl sulfate polyacrylamide gel electrophoresis and transferred onto a polyvinylidene fluoride membrane. The membrane was blocked with 5% (*w*/*v*) non-fat dry milk in PBS, and incubated with the desired primary antibody overnight at 4 °C. It was then washed and treated with the appropriate secondary antibody for 1 h at room temperature. Immunoreactive bands were detected using an enhanced chemiluminescence reagent (Thermo Scientific, Waltham, MA, USA). The blots were stripped and re-probed with an anti-actin or anti-GAPDH antibody, as appropriate, to normalize for differences in protein loading. Immunoreactive band intensity was determined after densitometric scanning, using Quantity One software (BioRad, Hercules, CA, USA) to quantify protein levels. For each protein, densitometric data were corrected for the loading difference and presented as a fold-change from the reference, control WT MEF, with the exception to LC3-II protein. In case of LC3-II, to make the measure of autophagic flux independent from differences in total LC3 level between cell lines (WT and S6K DKO MEF), the LC3-II level in cells under different autophagy-inducing conditions and ChQ in each line served as separate reference. The experiments were performed in triplicate. 

### 2.6. Immunofluorescence 

Cells were plated on coverslips, incubated overnight, and treated with 20 µM SFN for 6 h, glucose-deprived for 6 h, or serum-deprived for 24 h. Control cells were treated with a vehicle (DMSO) or grown in a complete (FBS- and glucose-supplemented) medium, accordingly. After treatment, the cells were washed with warm PBS and fixed at 37 °C for 15 min in 2% (*w*/*v*) paraformaldehyde in PBS. The cells were rinsed with PBS and permeabilized with 0.1% (*v*/*v*) Triton X-100 in PBS for 15 min. After washing five times with PBS, the cells were blocked in buffer containing 0.5% bovine serum albumin (BSA) and 0.15% glycine in PBS for 1 h, and incubated with anti-acetylated tubulin or anti- α-tubulin antibodies overnight. They were then washed three times with PBS and incubated with anti-mouse secondary antibodies conjugated to TRITC. After three washes with PBS, the cells were stained with 4′,6-diamidino-2-phenylindole (DAPI; Sigma-Aldrich, Saint Louis, MO, USA), washed with PBS (three times), mounted, and analyzed under DMI4000B inverted fluorescence microscope with a 100× objective (Leica Microsystems, Wetzlar, Germany). The acquired images were pseudocolored blue for DAPI and red for TRITC. The indicated sections of original images were enlarged, and deconvoluted and transformed into inverted grey scale using ImageJ software [35]. Approximately 100 cells from each sample were counted and classified as cells with either normal or abnormal cytoskeleton morphology. The experiments were performed in triplicate. 

### 2.7. Cathepsin L Activity Assay

Cells were grown and treated as indicated. They were then collected, washed in PBS, and processed using a Cathepsin L Activity Fluorometric Assay kit (BioVision, Milpitas, CA, USA; #K142), according to the manufacturer’s instructions. The cells were lysed in 55 µL of CL Cell Lysis Buffer for 10 min on ice and centrifuged at 4 °C at a maximum speed for 5 min. Then, 50 µL of supernatant (containing intact lysosomes) was transferred to the well of a 96-well plate. The remainder of the lysate was assayed by Bradford assay for protein load normalization. A well containing the CL Cell Lysis Buffer instead of the lysate served as a background control. Then, 50 µL of CL Reaction Buffer and 2 µL of Ac-FR-AFC Substrate (cathepsin-L substrate sequence FR labeled with AFC (amino-4-trifluoromethyl coumarin)) were added to each well. The plate was incubated at 37 °C for 1 h. The fluorescence of released free AFC (λ_Ex_/λ_Em_ = 400/505 nm) was determined using a microplate reader (Enspire Multimode Plate Reader, Perkin Elmer, Waltham, MA, USA). The experiments were performed in triplicate.

### 2.8. Flow Cytometry

WT MEF and S6K DKO MEF stably transfected with mCherry-GFP-LC3–encoding plasmid were grown in 6-well plates and treated for 16 h with 20 µM SFN or the vehicle (DMSO), or were grown in a growth factor-deprived or a control medium for 24 h. The cells were then collected by trypsinization, washed with warm PBS, and immediately analyzed using cytometer (Cube 6 Partec, Sysmex Europe Gmbh, Norderstedt, Germany) to measure the red and green fluorescence intensities. The experiments were performed in triplicate.

### 2.9. pH Measurements Using LysoSensor Yellow/Blue

Cells were grown in 12-well plates and treated with SFN or were serum-deprived. The cells were then collected, washed with warm Hank’s Balanced Salt Solution (HBSS), and stained for 5 min with 5 µM LysoSensor Yellow/Blue in HBSS. After additional washing, the cells were transferred to wells of a 96-well plate. Fluorescence intensities (I, λ_Ex_/λ_Em_ = 340/520 nm, and II, λ_Ex_/λ_Em_ = 380/520 nm) were measured using EnSpire Multimode plate reader (Perkin Elmer, Waltham, MA, USA) at 37 °C. Fluorescence intensity ratio (I/II) was then calculated and lysosomal pH was determined using a calibration curve. The experiments were performed in triplicate.

### 2.10. Statistical Analysis

Data were analyzed using GraphPad Prism (GraphPad Software, San Diego, CA, USA). One-way ANOVA followed by Dunnett’s or Bonferroni’s multiple comparison test was used to determine statistical significance of the differences in measured variables in tested groups. Differences were considered significant at *p* ˂ 0.05. 

## 3. Results

### 3.1. Impaired Autophagic Flux under Stress Conditions Is a General Phenotype of S6K DKO MEF

**Figure 1:**
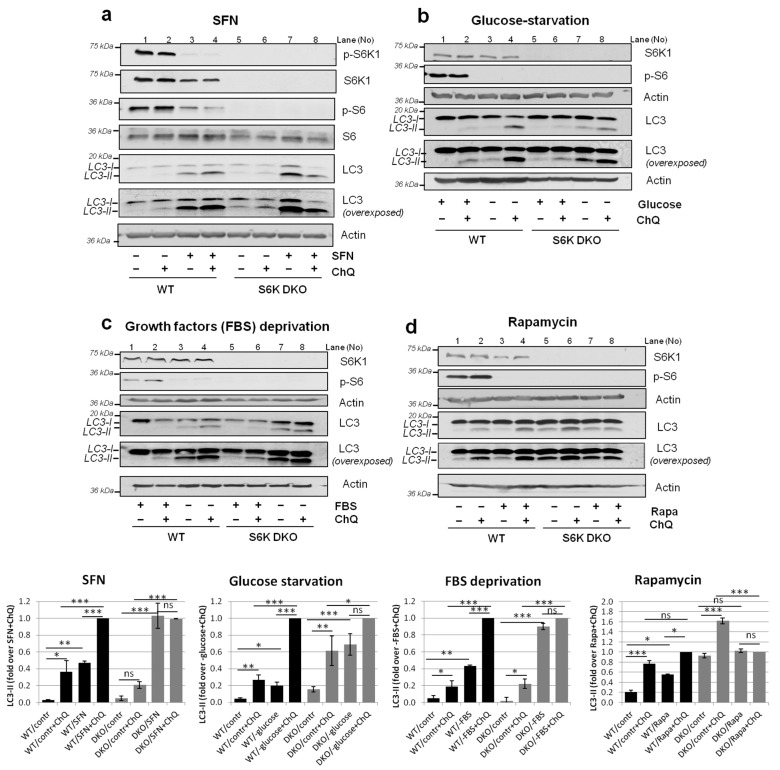
Impaired autophagic flux under stress conditions is a general phenotype of S6K DKO MEF. WT and S6K DKO MEF were grown under basal conditions: in a medium with a vehicle (DMSO; **a**,**d**) or a complete medium (**b**,**c**). Alternatively the cells were exposed to stress, by culturing in a medium deprived of either glucose (for 3 h; **b**) or growth factors (for 16–24 h; **c**); by a 16-h treatment with 20 µM sulforaphane (SFN; **a**); or a 3-h treatment with 100 nM rapamycin (Rapa; **d**). To assess the autophagic flux, a lysosomal inhibitor, chloroquine (ChQ; 10 µM), was added for the last 2 h of treatment, as indicated. The LC3, p-S6K1 (Thr389), S6K1, p-S6, and S6 protein levels were evaluated by western blotting. After the initial detection, the blots were re-probed with anti-β-actin antibodies to verify equal protein loading. Graphs below immunoblots show densitometric data, corrected for the loading difference and presented as a fold-change from the reference, WT or S6K DKO MEF under different autophagy-inducing conditions and ChQ (*lanes 4 and 8*, respectively). The experiment was performed in three independent replicates. The data are shown as the mean ± SEM. The statistical significance was determined by one-way ANOVA followed by Bonferroni’s multiple comparison test: * *p* < 0.05, ** *p* < 0.01, *** *p* < 0.001; ns, not significant.

Decreased S6K1 expression results in S6K2 overactivation, as a compensatory mechanism [21]. To avoid this confounding effect, in the current study, we used S6K1/2 double-knockout mouse embryonic fibroblasts (S6K DKO MEF), as we have previously demonstrated that S6K2 does not participate in autophagy regulation [30]. For each experiment, cell line identity was confirmed by immunoblotting of S6K1 (Figure 1a; S6K1 blots are omitted in the ensuing figures).

During autophagy, cytosolic LC3-I protein is modified, forming LC3-II, which is then incorporated into the autophagosome membrane. Consequently, increased LC3-II levels usually indicate enhanced autophagosome formation. However, it can also reflect autophagosome accumulation, caused by defective autophagosome degradation by lysosomes (impaired autophagic flux). To distinguish between these two scenarios, LC3-II levels in cells are compared in the presence or absence of a lysosomal inhibitor, e.g., chloroquine. Similar LC3-II levels in the presence or absence of chloroquine are indicative of an impaired autophagic flux. By contrast, increased LC3-II levels in the presence of chloroquine compared with those in untreated cells indicate normal autophagic flux. 

We have previously shown that 3-h SFN exposure efficiently induces autophagic flux in WT MEF, as assessed by comparing LC3-II levels in cells cultured in the presence or absence of chloroquine [30]. However, in analogously treated S6K DKO MEF, SFN-induced autophagic flux is impaired, indicating that S6K1 is indispensable for correct autophagosome degradation (we have excluded the role of S6K2 in autophagy flux in these analyses) [30]. In the current study, we first asked whether the lack of autophagic flux in S6K DKO MEF after 3-h SFN treatment was transient, e.g., caused by a delay in the removal of autophagosomes by lysosomes, or permanent, i.e., independent of the duration of SFN exposure. To test this, we treated WT and S6K DKO MEF with SFN for 16 h. As anticipated [30], SFN treatment led to a significant downregulation of p-S6K1 and p-S6 protein levels (Figure 1a). Concomitantly, we observed that, in contrast to WT cells, chloroquine did not elevate LC3-II levels in SFN-treated S6K DKO MEF, indicating a permanent block of autophagic flux in these cells (Figure 1a). 

Next, we asked whether the impact of S6K1 on autophagy is restricted only to SFN-induced autophagy or whether it is a general mechanism. To address this, we used other, well-documented autophagy inducers, namely, rapamycin, and glucose or growth factor deprivation [36,37,38,39]. First, we evaluated the effect of these factors on autophagic flux and S6K activity in both WT and S6K DKO MEF (Figure 1b–d). In WT cells, phosphorylation of the S6 ribosomal protein, evident under control conditions, became negligible upon autophagy stimulation, indicating effective inhibition of S6K activity by the applied stressors. Furthermore, in WT MEF, we observed the autophagic flux under both, basal and autophagy-stimulating (stress) conditions, as determined by an increase of LC3-II levels in the presence of chloroquine. Notably, in S6K DKO MEF, the autophagic flux was observed only under basal conditions and was abrogated (or diminished) under stress (since rapamycin did not effectively induce autophagy in S6K DKO MEF, we did not use it in subsequent experiments). However, the absence of autophagy flux in stressed S6K DKO MEF did not result from defects in autophagy induction since the levels of lysosomal and autophagy-related proteins in WT and S6K DKO MEF were similar (of even higher in S6K DKO MEF), both in control and stress conditions (Supplementary Figure S1). Moreover, the immunofluorescence assay indicated the efficient formation LC3-positive vacuoles (i.e., autophagosomes) and their increased accumulation in SFN-treated or growth factors-deprived S6K DKO MEF compared to their WT counterparts (Supplementary Figure S2). 

### 3.2. Absence of S6K Kinases Does Not Perturb Lysosomal Function under SFN-Induced Stress

**Figure 2:**
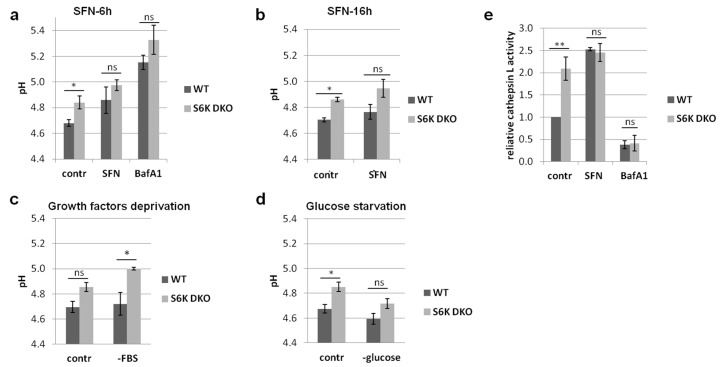
The absence of S6K kinases does not perturb lysosomal function under stress conditions. The impact of S6K status on lysosomal pH (**a**–**d**) and cathepsin L activity (**e**) was analyzed. WT and S6K DKO MEF were treated with 20 µM SFN for 6 h (**a**), 16 h (**b**), or 3 h (**e**), or deprived of growth factors for 6 h (**c**) or glucose for 3 h (**d**). Control cells (contr) were treated with the vehicle (DMSO; **a**,**b**,**e**) or grown in a complete medium (**c**,**d**). Cathepsin L activity and pH were measured as described in Materials and Methods (Section 2.7 and Section 2.9, respectively). Cells treated with bafilomycin A1 (BafA1; 100 nM) served as a positive control. The data are shown as the mean ± SEM. Statistical significance was determined by one-way ANOVA followed by Bonferroni’s multiple comparison test: * *p* < 0.05, ** *p* < 0.01; ns, not significant. The experiments were performed in three independent replicates.

To determine whether the lack of lysosomal degradation of autophagosomes in S6K DKO MEF was caused by impaired lysosome function, we evaluated lysosomal pH by ratiometric measurements of fluorescence of a pH-sensitive dual-excitation and dual-emission lysosomal dye, LysoSensor Yellow/Blue. To do this, WT and S6K DKO MEF were treated with 20 μM SFN, the vehicle (DMSO), or 100 nM bafilomycin A1 serving as a positive control. As shown in Figure 2a, under the control conditions, the lysosomal pH in S6K DKO MEF was slightly higher than that in WT cells (pH 4.68 vs. 4.84 in WT or S6K DKO MEF, respectively). When exposed to SFN for 6 h, the lysosomal pH increased in both cell lines, reaching similar values (pH 4.86 vs. 4.98 in WT or S6K DKO MEF, respectively), while bafilomycin A1 treatment increased the lysosomal pH to 5.15 in WT cells and 5.33 in S6K DKO MEF. A prolonged SFN treatment (16 h) had no further impact on the pH (Figure 2b). The observed increase in lysosomal pH induced by SFN is in an agreement with a report of Wilcox et al. [40] that SFN elevates pH of the yeast vacuole. 

We also determined the impact of growth factor (FBS-) deprivation and glucose starvation on the lysosomal pH. A 6-h growth factor deprivation resulted in a slight increase of lysosomal pH; however, it did not exceeded pH 5.0 (Figure 2c). A prolonged treatment did not affect the pH further (data not shown). On the other hand, glucose starvation decreased the pH in both cell lines (Figure 2d). The increase of lysosomal pH by serum deprivation and its decrease induced by glucose starvation are in agreement with other studies [41,42].

Due to the increase in lysosomal pH may affect the activity of acidic hydrolases, we asked whether the reduced LC3 degradation in S6K DKO MEF was caused by a decreased activity of lysosomal enzymes. To address this, we measured enzymatic activity of cathepsin L, a lysosomal protease responsible for the degradation of LC3-II protein, with an activity optimum at pH 5.0 [43,44]. Unexpectedly, under the control conditions, the relative activity of cathepsin L in S6K DKO MEF was almost two-fold higher than that in WT MEF (Figure 2e). However, upon SFN-induced stress, it was the same in both cell lines (2.5-fold higher than that in WT MEF under the control conditions). Bafilomycin A1 treatment decreased cathepsin L activity nearly two-fold in both cell lines (Figure 2e). Collectively, blocked autophagic flux in stressed S6K DKO MEF is not caused by an impaired activity of lysosomal enzymes.

### 3.3. Autophagosome-Lysosome Fusion Is Impaired in S6K DKO MEF under Stress Conditions

**Figure 3:**
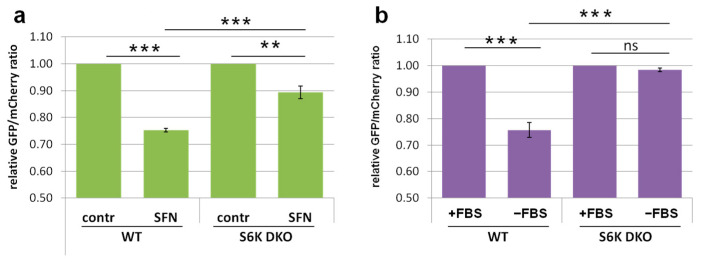
Lack of S6K kinases affects autophagosome-lysosome fusion in cells stressed by SFN exposure or by growth factor deprivation. (**a**) Impact of 20 µM SFN (6 h) or (**b**) growth factor deprivation (−FBS; 24 h) on GFP/mCherry fluorescence intensity ratio in WT or S6K DKO MEF stably overexpressing GFP-mCherry-LC3, (as described in Materials and Methods). Control cells (contr) were treated with the vehicle (DMSO; **a**) or were grown in FBS-supplemented medium (**b**). The experiment was performed in three independent replicates. The data are shown as the mean ± SEM. Statistical significance was determined by one-way ANOVA followed by Bonferroni’s multiple comparison test: ** *p* < 0.01, *** *p* < 0.001; ns, not significant.

Reduced autophagosome maturation in S6K DKO MEF might have been a result of perturbed autophagosome-lysosome fusion. To test this scenario, we took advantage of WT and S6K DKO MEF stably expressing mCherry- and GFP-tagged LC3 (mCherry-GFP-LC3), a useful tool for studying autophagosome maturation. In this approach, at neutral pH in the autophagosome, both LC3 tags emit fluorescence. However, after fusion of the autophagosome with the lysosome, the acidic autolysosomal environment quenches GFP fluorescence, whereas pH-insensitive mCherry fluorescence is preserved [34,45]. Therefore, a decreased GFP/mCherry fluorescence intensity ratio is a measure of autolysosome formation. We treated WT MEF and S6K DKO MEF stably expressing mCherry-GFP-LC3 with SFN or the vehicle, and immediately measured the GFP/mCherry fluorescence intensity by flow cytometry. The results are presented in Figure 3a. In SFN-treated WT MEF, the GFP/mCherry fluorescence intensity ratio decreased by 25% compared to that in vehicle-treated cells, indicating efficient autolysosome formation. This effect was significantly reduced in S6K DKO MEF (an intensity ratio decrease by only 11%), suggesting that S6K1 is required for an appropriate fusion and maturation of autophagosomes into autolysosomes during cell stress. 

To test this hypothesis, we used another, well documented model of autophagy-inducing stress, i.e., growth factor deprivation [36,37,38,39]. In accordance with the results described above, in serum-deprived cells, the absence of S6K1 (we have previously excluded the role of S6K2 in autophagy) abolished the decrease in GFP/mCherry fluorescence intensity ratio observed in WT cells (Figure 3b). This confirms that the S6K1 kinase is necessary for the autophagosome-lysosome fusion not only during SFN-induced autophagy but also during stress induced by growth factor deprivation.

### 3.4. Lack of S6K1 Kinase Affects Acetylated Microtubules but Not Total Microtubule Network in Cells Exposed to Stress

**Figure 4:**
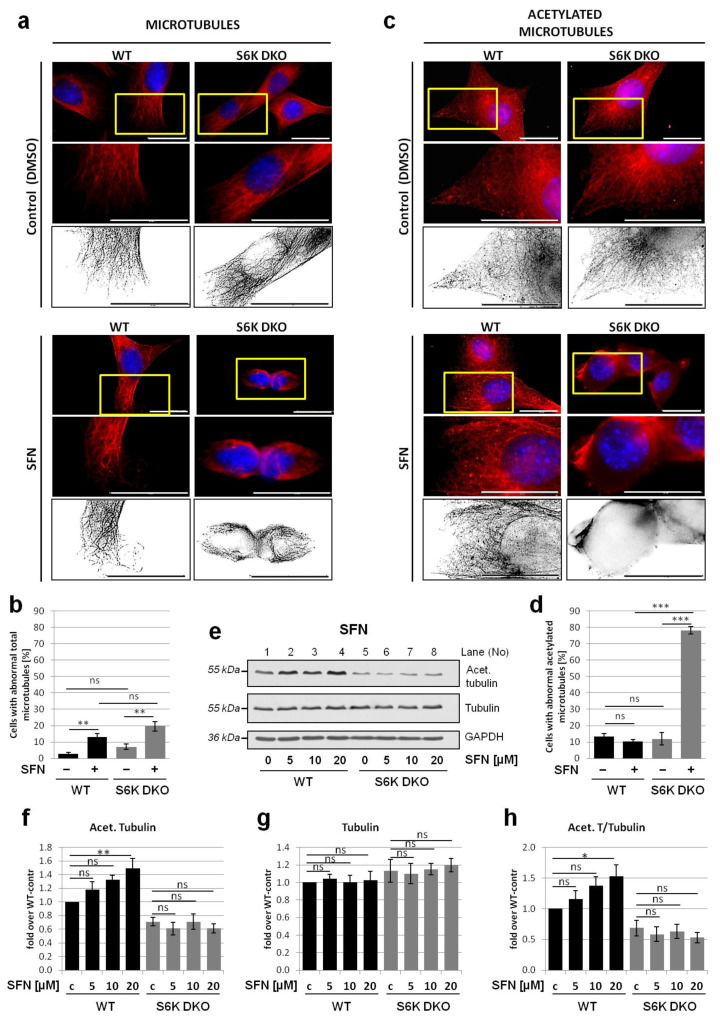
Acetylated microtubules but not the total microtubule network are disrupted in S6K DKO MEF treated with SFN. Total microtubule network (**a,b**) and acetylated microtubule network (**c,d**) were investigated by immunofluorescent staining of total α-tubulin (**a**) or acetylated tubulin (**b**), respectively, in WT and S6K DKO (DKO) MEF treated with 20 µM SFN or DMSO (control) for 6 h. The cells were stained with mouse antibodies against α-tubulin (**a**) or against acetylated tubulin (Lys40) (**b**), followed by staining with goat anti-mouse–TRITC conjugates. DNA was stained with DAPI (blue). The indicated sections of original images are enlarged (middle panels) and, for clarity, deconvoluted and transformed into inverted grey scale (bottom panels). Representative images of cells analyzed in at least 10 random fields per replicate are shown. Scale bar = 25 µm. Approximately 100 cells per sample were counted in three independent replicates. The percentage of cells with a diffused, depolymerized (abnormal) microtubule (**b**) and acetylated microtubule (**d**) network was counted and graphed (mean ± SEM). Statistical significance was determined by one-way ANOVA followed by Bonferroni’s multiple comparison test: ** *p* < 0.01, *** *p* < 0.001; ns, not significant. (**e**) Acetylated (Lys40) and total α-tubulin levels were assessed by western blotting in mouse WT and S6K DKO MEF exposed to 5, 10, and 20 µM SFN, or DMSO (control; **c**) for 3 h. The blots were re-probed with anti-GAPDH antibodies to ensure equal protein loading. Densitometric data of acetylated tubulin (**f**) and tubulin (**g**) levels corrected for loading control, as well as acetylated tubulin to total tubulin ratio (**h**) are presented as a fold-change from the reference, the control WT MEF (*lane 1*). The experiment was performed in three independent replicates. The data are shown as the mean ± SEM. The statistical significance of differences was determined by one-way ANOVA followed by Bonferroni’s multiple comparison test: * *p* < 0.05, ** *p* < 0.01, *** *p* < 0.001; ns, not significant.

The process of autophagosome and lysosome fusion requires intact, acetylated microtubules that enable the transport of autophagosome to the perinuclear region, where the lysosomes are located [7,14,46]. Enhanced microtubules acetylation (hyperacetylation) is a rapid and general cell response to various stresses, and protects the microtubules against depolymerization [7,12,47]. To investigate whether SFN affects the microtubule network, we examined the organization of total microtubule network, after immunostaining with anti-α-tubulin antibodies, and acetylated microtubule morphology, which were immunostained with antibodies recognizing only acetylated α-tubulin. Under basal conditions, the total microtubule network was clearly visible in both WT and S6K DKO MEF, and was preserved in both cell lines despite S6K DKO MEF shrinkage upon SFN treatment (Figure 4a,b).

The morphology of acetylated microtubule network was comparable in both cell lines under basal conditions; however, in S6K DKO MEF, the network was more delicate, less dense and the fluorescent signal was less intense. When the cells were exposed to SFN, the acetylated microtubule network remained intact only in WT MEF, and it was almost completely dispersed in S6K DKO MEF (Figure 4c,d).

To determine whether disruption of the acetylated microtubule network in SFN-treated S6K DKO MEF resulted from microtubule depolymerization or was a consequence of reduced amount of acetylated tubulin, we performed western blotting. Administration of increasing doses of SFN (5, 10, or 20 μM) to WT cells led to a proportional increase in acetylated tubulin levels. In S6K DKO MEF cultured under basal conditions, the acetylated tubulin levels were lower than those in WT cells and, in contrast to WT MEF, did not further increase after SFN exposure (Figure 4e,f,h). The amount of total α-tubulin was comparable in both cell lines, irrespective of treatment (Figure 4e,g). Collectively, this experiment demonstrates that the absence of S6K1 impairs SFN-induced hyperacetylation of tubulin. Since tubulin is acetylated only in polymerized microtubules (not in the soluble form), the immunoblotting data suggest a diminished number of acetylated microtubules in these cells during SFN-induced stress, confirming the results of immunofluorescence analysis. 

**Figure 5:**
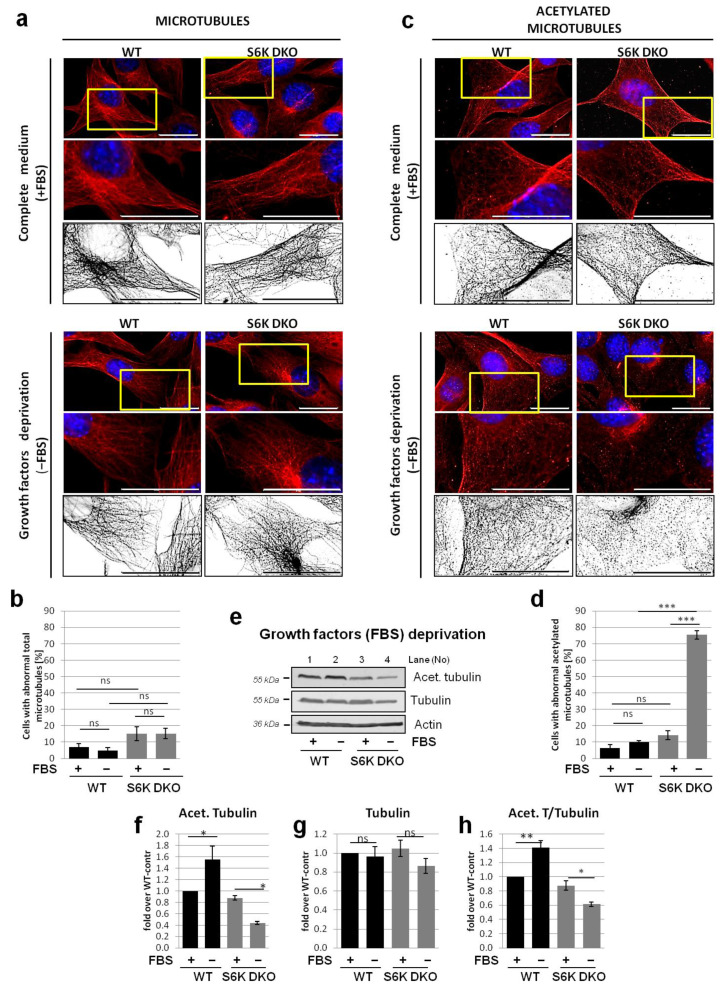
Lack of S6K affects the acetylated but not the total microtubule network in cells deprived of growth factors. Total (**a,b**) and acetylated microtubule network (**c,d**) was investigated by immunofluorescent staining of total α-tubulin (**a**) or acetylated tubulin (**b**), respectively, in WT and S6K DKO (DKO) MEF growing in a complete medium (control; +FBS) or deprived of growth factors (−FBS) for 16 h. The cells were stained using mouse antibodies against α-tubulin (**a**) or acetylated tubulin (Lys40) (**b**), followed by staining with goat anti-mouse–TRITC conjugates. DNA was stained with DAPI (blue). The indicated sections of original images are enlarged (middle panels), and for clarity, deconvoluted and transformed into inverted grey scale (bottom panels). Representative images of cells analyzed in at least 10 random fields per experiment are shown. Scale bar = 25 µm. Approximately 100 cells per sample were counted in three independent replicates. The percentage of cells with a diffused, depolymerized (abnormal) microtubule (**b**) and acetylated microtubule (**d**) network was counted and graphed (mean ± SEM). Statistical significance was determined by one-way ANOVA followed by Bonferroni’s multiple comparison test: ** *p* < 0.01, *** *p* < 0.001; ns, not significant. (**e**) Acetylated tubulin and total α-tubulin levels were evaluated by western blotting in WT and S6K DKO MEF grown in a complete medium (control; +FBS) or a medium deprived of growth factors (−FBS) for 16 h. The blots were re-probed with anti-β-actin antibodies to ensure equal protein loading. Densitometric data of acetylated tubulin (**f**) and tubulin (**g**) levels, corrected for loading control, as well as acetylated tubulin to total tubulin ratio (**h**) are presented as a fold-change from the reference, the control WT MEF (*lane 1*). The experiment was performed in three independent replicates. The data are shown as the mean ± SEM. The statistical significance of differences was determined by one-way ANOVA followed by Bonferroni’s multiple comparison test: * *p* < 0.05, ** *p* < 0.01, *** *p* < 0.001; ns, not significant.

We also performed an analogous experiment using growth factor deprivation as an autophagy-inducing stressor. Immunofluorescent staining of total α-tubulin revealed that in both WT and S6K DKO MEF, the total microtubule network remained intact under basal as well as serum-deprived conditions, although cells from both cell lines changed shape and flattened in the absence of growth factors (Figure 5a,b). Acetylated microtubule morphology was similar in WT and S6K DKO MEF cultured in the presence of serum (basal conditions) but the differences between cell lines became striking when the growth factors were omitted (Figure 5c,d). Compared to WT cells, the acetylated microtubules in S6K DKO MEF became less abundant, shorter, and disrupted. Furthermore, we noticed enhanced staining in the centrosomal nucleation regions [48]; the staining was also observed in stressed WT MEF but was not as pronounced. 

We also compared acetylated and total α-tubulin levels in cells stressed by growth factor (serum) deprivation. Immunoblotting analysis revealed that whereas total α-tubulin levels were similar in both cell lines, the acetylation of tubulin increased in WT MEF but decreased in S6K DKO MEF during stress associated with the lack of growth factors (Figure 5e,h). 

**Figure 6:**
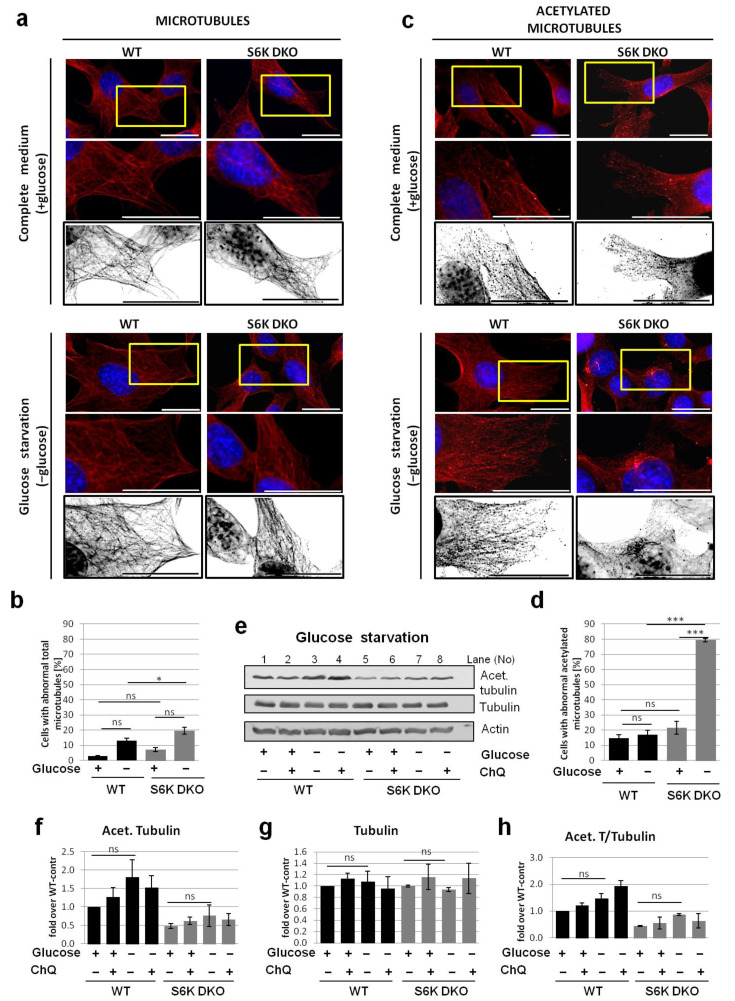
Lack of S6K affects the acetylated but not the total microtubule network in glucose-starved cells. Total (**a,b**) and acetylated (**c,d**) microtubule network was investigated by immunofluorescent staining of total α-tubulin (**a**) or acetylated tubulin (**b**), respectively, in WT and S6K DKO (DKO) MEF grown in a complete medium (control; +glucose) or deprived of glucose (−glucose) for 6 h. The cells were stained using mouse antibodies against α-tubulin (**a**) or acetylated tubulin (Lys40) (**b**), followed by staining with goat anti-mouse–TRITC conjugates. DNA was stained with DAPI (blue). The indicated sections of original images are shown enlarged (middle panels) and, for clarity, deconvoluted and transformed into inverted grey scale (bottom panels). Representative images of cells analyzed in at least 10 random fields per experiment are shown. Scale bar = 25 µm. Approximately 100 cells per sample were counted in three independent replicates. The percentage of cells with a diffused, depolymerized (abnormal) microtubule (**b**) and acetylated microtubule (**d**) network was counted and graphed (mean ± SEM). Statistical significance was determined by one-way ANOVA followed by Bonferroni’s multiple comparison test: *** *p* < 0.001; ns, not significant. (**e**) Acetylated tubulin and total α-tubulin levels were evaluated by western blotting in WT and S6K DKO MEF grown in a complete medium (control; +glucose) or a medium deprived of glucose for 3 h (−glucose). The blots were re-probed with anti-β-actin antibodies to ensure equal protein loading. Densitometric data, of acetylated tubulin (**f**) and tubulin (**g**) levels, corrected for loading control, as well as acetylated tubulin to total tubulin ratio (**h**) are presented as a fold-change from the reference, the control WT MEF (*lane 1*). The experiment was performed in three independent replicates. The data are shown as the mean ± SEM. The statistical significance of differences was determined by one-way ANOVA followed by Bonferroni’s multiple comparison test: * *p* < 0.05, *** *p* < 0.001; ns, not significant.

The impact of another stressor, glucose starvation, on microtubule morphology was also assessed (Figure 6). Similarly to serum-deprived cells, we did not observe significant differences in total microtubule morphology (Figure 6a,b). In the case of acetylated microtubules, the cytoskeleton was more delicate and less dense in S6K DKO MEF under basal conditions, but did not differ significantly from that observed in WT MEF (Figure 6c,d). However, when the cells were glucose-starved, the acetylated cytoskeleton density increased in WT cells, while it was negligent in S6K DKO MEF. Further, glucose starvation resulted in a notable (although not statistically significant) increase in tubulin acetylation in WT MEF but not in S6K DKO MEF (Figure 6e,h). The presence of chloroquine did not affect tubulin acetylation. 

Collectively, the above experiments demonstrate that the acetylated microtubule network, and not the total microtubule network, is affected by the lack of S6K1 exclusively under stress (but not basal) conditions. They also support the hypothesis that the impact of S6K1 on *s*tress-mediated tubulin acetylation, and thus, autophagic flux, is a mechanism that is conserved and independent of the autophagy-inducing stimuli.

### 3.5. Overexpression of Exogenous S6K1 Restores Tubulin Acetylation and Lysosomal LC3-II Degradation in S6K DKO MEF Exposed to SFN

**Figure 7:**
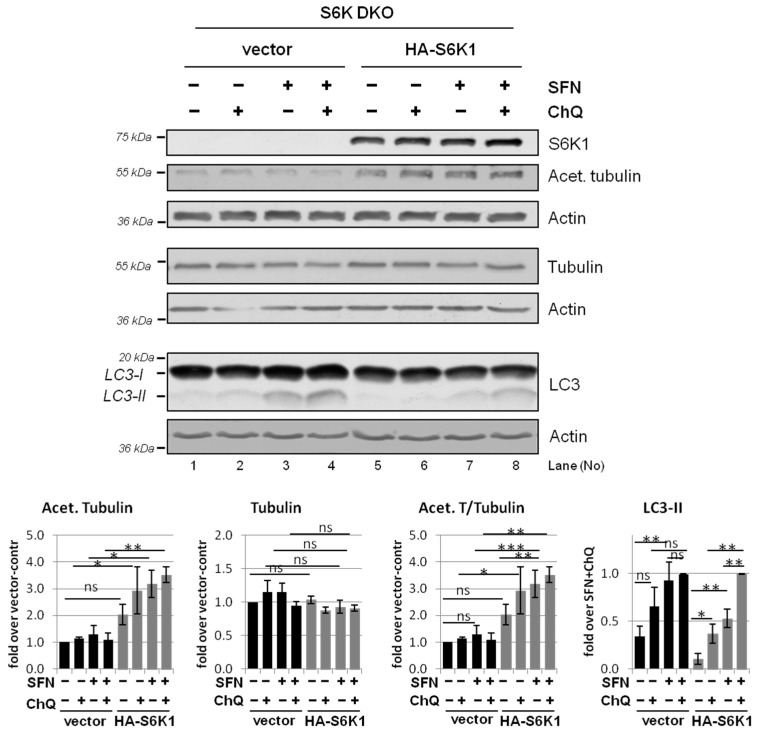
Overexpression of exogenous S6K1 restores tubulin acetylation and LC3-II degradation in S6K DKO MEF exposed to SFN. S6K DKO MEF were transfected with a plasmid coding for HA-S6K1 or a control vector, and exposed to 20 µM SFN or DMSO (control) for 3 h. The lysosomal inhibitor chloroquine (ChQ; 10 µM) was added for the last 2 h of treatment, as indicated. The S6K1, acetylated tubulin, total α-tubulin, and LC3 levels were evaluated by western blotting. The blots were re-probed with anti-β-actin antibodies to ensure equal protein loading. Graphs below immunoblots show densitometric data, corrected for loading control and presented as a fold-change from the reference, i.e., cells transfected with the control vector and treated with DMSO (*lane 1*) for S6K1, acetylated tubulin, and total α-tubulin, and cells transfected with the control or HA-S6K1 encoding vector and treated with SFN and ChQ (*lane 4* and *8*, respectively) for LC3-II. The experiment was performed in three independent replicates. The data are shown as the mean ± SEM. The statistical significance of differences was determined by one-way ANOVA followed by Bonferroni’s multiple comparison test: * *p* < 0.05, ** *p* < 0.01, *** *p* < 0.001; ns, not significant.

To confirm the unexpected role of S6K1 in the control of tubulin acetylation (and, in turn, autophagic flux), we performed rescue experiments by transiently transfecting S6K DKO MEF with an S6K1-encoding plasmid (the impact of S6K2 on autophagy was previously excluded [30]). To evaluate the autophagic flux, the cells were treated with SFN or the vehicle in the presence or absence of chloroquine. Indeed, reintroduction of S6K1 improved autophagic flux in SFN-treated S6K DKO MEF (Figure 7 and [30]). This was accompanied by an increase of acetylated tubulin levels, particularly in SFN-treated cells. This effect was not apparent in cells transfected with the control vector, confirming that S6K1 plays an essential role in the regulation of tubulin acetylation and is necessary for its enhancement under stress conditions.

### 3.6. Modulation of Tubulin Acetylation by S6K1 Is Conserved in Different Cells

**Figure 8:**
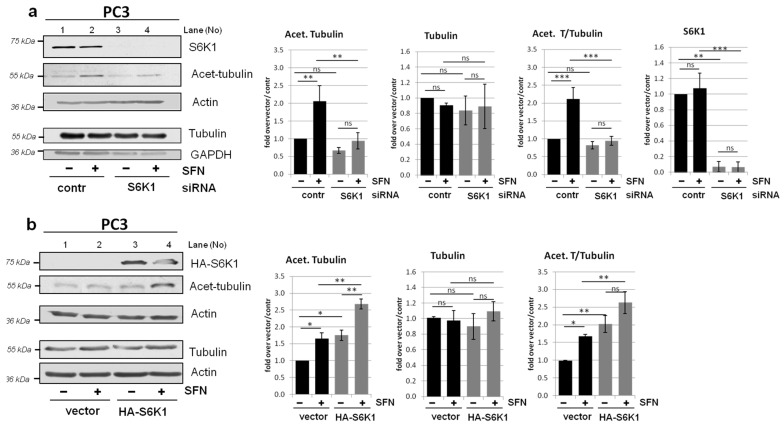
Modulation of tubulin acetylation by S6K1 is conserved in different cellular models. Prostate cancer cells (PC3) were transfected with (**a**) a control or S6K1-targeting siRNA, or (**b**) a control or HA-S6K1–encoding vector (**b**). The cells were treated with DMSO (control) or SFN for 3 h. Acetylated tubulin, S6K1, and HA-S6K1 protein levels were evaluated by western blotting. The blots were re-probed with anti-β-actin antibodies to ensure equal protein loading. Graphs next to immunoblots show densitometric data, corrected for loading control and presented as fold-changes from the reference, untreated control cells (*lane 1*). The experiment was performed in three independent replicates. The data are shown as the mean ± SEM. The statistical significance of differences was determined by one-way ANOVA followed by Bonferroni’s multiple comparison test: * *p* < 0.05, ** *p* < 0.01, *** *p* < 0.001; ns, not significant.

To address the question of whether the role of S6K1 in tubulin acetylation and autophagic flux is cell type-specific or a conserved mechanism, we investigated another cellular model, PC3 prostate cancer cells. We examined the impact of S6K1 status on tubulin acetylation by silencing *S6K1* gene expression using a pool of three target-specific siRNAs, and overexpressed the gene by transient transfection with a plasmid encoding HA-tagged S6K1. As shown in Figure 8a, downregulation of *S6K1* efficiently decreased the levels of acetylated tubulin in PC3 cells. On the other hand, overexpression of *S6K1* increased the acetylation of tubulin, particularly in SFN-treated cells (Figure 8b). These observations support the notion of a direct and indispensable role of S6K1 in tubulin acetylation.

### 3.7. Role of S6K1 in Tubulin Acetylation Is Regulated by Its Phosphorylation Status

**Figure 9:**
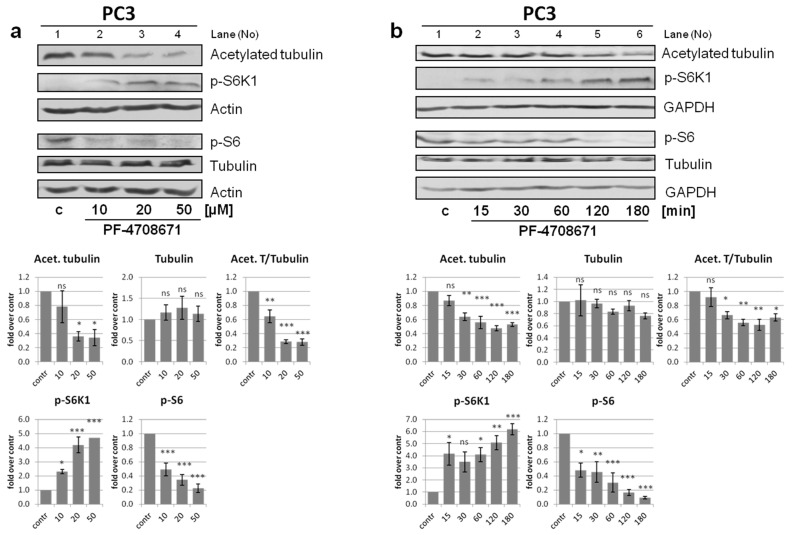
Modulation of tubulin acetylation by S6K1 is dependent on its phosphorylation status. Prostate cancer cells (PC3) were treated (**a**) for 3 h with the indicated concentrations of PF-4708671, or (**b**) with 10 µM PF-4708671 for the indicated time periods. The control cells (**c**) were treated with the vehicle (DMSO). Acetylated tubulin, tubulin, p-S6K1 (Thr389), and p-S6 protein levels were evaluated by western blotting. The blots were re-probed with anti-β-actin or anti-GAPDH antibodies to ensure equal protein loading. Graphs below immunoblots show densitometric data, corrected for loading control and presented as a fold-change from the reference (control; *line 1*). The experiments were performed in three independent replicates and representative blots are shown. The data are shown as the mean ± SEM. The statistical significance of differences between control and treated cells was determined by one-way ANOVA followed by Dunnett’s multiple comparison test: * *p* < 0.05, ** *p* < 0.01, *** *p* < 0.001; ns, not significant.

During the SFN treatment, S6K1 is concomitantly catalytically inactivated and dephosphorylated at Thr389 (Figure 9a). Phosphorylation of this residue is not only essential for S6K1 kinase activity but also regulates the protein’s nucleocytoplasmic localization [49]. This prompted us to ask whether the inactivation of kinase activity is the only factor responsible for the role of S6K1 in tubulin acetylation, i.e., whether its phosphorylation status also plays a role. To address this, we took advantage of PF-4708671, an S6K1 inhibitor, which blocks S6K1 kinase activity despite enhancing its phosphorylation at Thr389 [49,50]. PF-4708671 allowed us to separate S6K1 activity from the protein’s phosphorylation status. 

We treated PC3 cells with increasing doses of the inhibitor for 3 h (Figure 9a), or with 10 µM PF-4708671 for different time periods (Figure 9b). The activity of S6K1 was efficiently inhibited, manifested as a gradual reduction in the phosphorylation of its substrate, the S6 protein. Concomitantly, we observed a gradual increase in S6K1 phosphorylation, as anticipated [49,50]. Notably, S6K1 inhibition and concomitant increase in Thr389 phosphorylation was accompanied by a dose- and time-dependent decrease in the acetylated tubulin levels. This indicates that an increased Thr389 phosphorylation disrupts the ability of inactive S6K1 to mediate tubulin acetylation. Further, since PF-4708671 is a highly specific inhibitor of S6K1 but not S6K2 (IC_50_: 0.16 µM vs. 65 µM, respectively), while having no effect on the closely related RSK and MSK kinases [50], these observations additionally support our previous findings that S6K1, but not S6K2, is a principal kinase regulating the autophagic flux [30].

### 3.8. Overexpression of Acetylation-Mimicking but Not Acetylation-Resistant Tubulin Variant Restores Autophagic Flux in S6K DKO MEF

**Figure 10:**
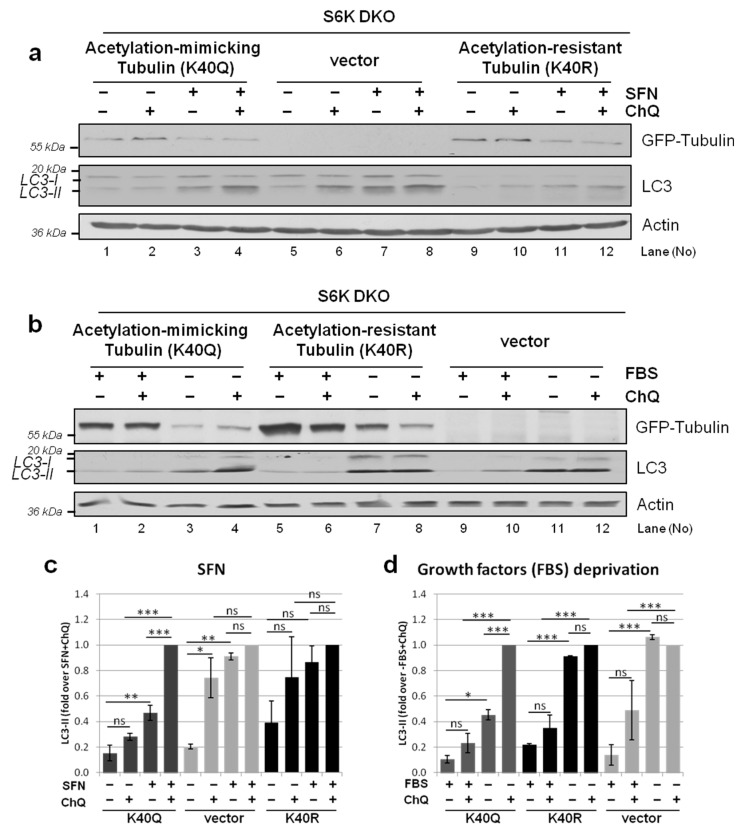
Overexpression of an acetylation-mimicking but not acetylation-resistant variant of tubulin restores autophagic flux in stressed S6K DKO MEF. The impact of the overexpression of acetylation-mimicking (K40Q) or acetylation-resistant (K40R) GFP-tubulin variants on autophagic flux during stress induced by SFN (20 µM; 3 h) (**a,c**) or growth factor deprivation (−FBS; 24 h) (**b,d**) in S6K DKO MEF was evaluated by immunoblotting. Cells transfected with an empty vector served as a control. To assess the autophagic flux, chloroquine (ChQ; 10 µM), a lysosomal inhibitor, was added for the last 2 h of treatment, as indicated. GFP-tubulin and LC3 protein levels were evaluated by western blotting. The blots were re-probed with anti-β-actin antibodies to ensure equal protein loading. Densitometric data, corrected for loading control, are shown as a fold-change from the reference, i.e., the cells transfected with the control, acetylation-mimicking (K40Q) or acetylation-resistant (K40R) GFP-tubulin vector, and treated with SFN + ChQ *(lane 8*, *4* and *12*, respectively) (**a,c**) or deprived of FBS + ChQ (*lane 12*, *4* and *8*, respectively) (**b,d**). The experiments were performed in three independent replicates. The data are shown as the mean ± SEM. The statistical significance of differences was determined by one-way ANOVA followed by Bonferroni’s multiple comparison test: * *p* < 0.05; ** *p* < 0.01, *** *p* < 0.001; ns, not significant.

To unequivocally verify the hypothesis that disturbed autophagic flux in S6K1-deficient cells results from impaired tubulin acetylation and not changes in the tubulin levels *per se*, we transfected S6K DKO MEF with a control vector or a vector coding for GFP-tagged α-tubulin acetylation-mimicking (K40Q) or acetylation-resistant (K40R) variants [33,51,52,53,54,55]. We first used fluorescence microscopy to confirm that the variants are effectively incorporated into the microtubule cytoskeleton (data not shown). We then assessed their impact on the flux of autophagy induced by either SFN treatment or growth factor deprivation. As shown in Figure 10, in S6K DKO MEF transfected with an empty vector, the autophagic flux was present under the basal conditions, but not upon autophagy induced by either SFN (Figure 10a,c) or serum deprivation (Figure 10b,d). By contrast, overexpression of the acetylation-mimicking tubulin variant effi-ciently restored autophagic flux in stressed S6K DKO MEF. Since this effect was not observed in cells transfected with the acetylation-resistant tubulin variant, the experiment confirmed the pivotal role of tubulin acetylation in the restoration of stress-induced autophagic flux in S6K DKO MEF. 

## 4. Discussion

While it is known that mTORC1 is the main negative regulator of autophagy, the role of its direct substrate, S6K1, in this process, is not clear. In the current study, using an array of molecular biology tools in different cell models, we showed that S6K1 is crucial for proper autophagosome degradation by controlling the process of acetylation of microtubules—a key step for autophagosome-lysosome fusion. This finding points to a new, kinase-independent role of S6K1 and could shed new light on the pathomechanism of disorders associated with dysregulation of S6K1 and autophagy, such as obesity, insulin resistance, neurodegenerative diseases, myopathies, and cancer [3,19,21,22,23,24].

Many contradictory conclusions have been drawn from studies focusing on the role of S6K1 in autophagy [25,26,28]. However, these studies were performed in cellular models originating from different species. Further, modulation of S6K1 activity therein was achieved using various approaches, such as inhibition of protein activity (by overexpression of a kinase-dead variant, or the application of S6K1 or mTORC1 inhibitors), reduction of protein levels (by siRNA or a genetic knockout), as well as induction of constitutive activity (by overexpression of relevant S6K1 mutants). Notably, none of these studies focused on the role of S6K1 in the final stages of autophagy. Further, since various experimental conditions were used therein, some studies in effect investigated basal autophagy, while others investigated stress-induced autophagy. Therefore, the role of S6K1 in autophagy remained unresolved.

We have previously provided evidence that S6K1 is essential for autophagic flux in stress-induced but not basal autophagy. However, the precise mechanism of this phenomenon remained elusive [30]. Impaired autophagosome clearance can result from a decreased lysosomal activity or impaired autophagosome trafficking towards the lysosome (required for their fusion). We here tested both these hypotheses, to clarify which of the mechanisms block autophagosome degradation in S6K DKO MEF. The presented data indicate that this block is not caused by the inhibition of lysosomal function but results from diminished tubulin acetylation and, in turn, defective autophagosome-lysosome fusion. Five lines of evidence support this conclusion. First, although it was noted that the relatively elevated lysosomal pH inhibits the autophagosome-lysosome fusion [56], it is unlikely to be the primary reason for the lack of autophagosome degradation in this case because the extent to which the lysosomal pH was elevated during SFN treatment in both cell lines tested was similar and not high enough to inhibit cathepsin L, an acidic lysosomal hydrolase responsible for LC3-II degradation [57]. Second, the observed increased GFP/mCherry fluorescence ratio in S6K DKO MEF compared with WT MEF under stress conditions cannot be interpreted to mean an increase in lysosomal pH instead of a block of the autophagosome-lysosome fusion, as the pH in both cell lines was not higher than 5, and thus GFP fluorescence was efficiently quenched [58,59,60]. Third, we have previously demonstrated that during SFN-induced autophagy in S6K DKO MEF or S6K1-depleted PC3 cells, the number of autolysosomes is decreased while that of autophagosomes is elevated which indicates that autophagosome-lysosome fusion is blocked if S6K1 is missing [30]. Fourth, disruption of the microtubule network affects lysosomal function [61,62,63], which implies that the altered lysosome phenotype in S6K DKO MEF is a consequence, rather that the underlying reason, of an impaired microtubule network. Fifth, overexpression of an acetylation-mimicking tubulin variant in stressed S6K DKO MEF efficiently restored autophagic flux, whereas that of acetylation-resistant variant failed to do so. These findings strongly support the hypothesis that restoration of tubulin acetylation is essential for the rescue of the S6K DKO MEF phenotype. In the current study, we showed for the first time that S6K1 controls microtubule acetylation under stress conditions and, consequently, the process of autophagosome-lysosome fusion. Furthermore, the control of tubulin acetylation by S6K1 seems to be a general mechanism, as it was indispensable during autophagy mediated by very different stressors, i.e., SFN, and glucose or growth factor deprivation. Finally, we here confirmed the effects of SFN and growth factor deprivation in both cancerous and non-cancerous cells.

One of the purposes of the current study was to address the question of why S6K1 is needed only under stress conditions and the underlying mechanism. An increasing body of evidence suggests that microtubule acetylation is an important link between stress and autophagy [7,14,64]. Under standard conditions (basal autophagy), the two microtubule motor proteins, kinesin (anterograde intracellular transport) and dynein (retrograde intracellular transport), participate in autophagosome trafficking toward the lysosome. However, under stress conditions, the only motor protein responsible for autophagosome transport and the subsequent autophagosome-lysosome fusion is dynein [14,15,65]. This might be related to the observation that, upon stress, lysosomes concentrate in the perinuclear region of the cell [66]. Of note, binding of dynein to microtubules is increased upon microtubule acetylation, while increased acetylation is a general cell response to stress ([7,14] and Figure 4, Figure 5 and Figure 6). Furthermore, inhibition of dynein under stress conditions results in an impaired autophagic flux [14,15,65,67]. Data presented in the current study indicate that cellular S6K1 deficiency disturbs the process of microtubule acetylation. This, in consequence, might block the binding of dynein to microtubules and inhibit autophagosome trafficking to the lysosome. Since S6K1 affects microtubule acetylation only under stress conditions, this might explain why the absence of S6K1 under standard, basal, conditions does not interfere with the autophagic flux. 

Another possible explanation for the impaired autophagic flux observed in S6K DKO MEF is that the lack of S6K affects autophagic lysosome reformation (ALR). ALR is a terminal step of autophagy, during which a long tubular structure extends from an autolysosome and a vesicle, the proto-lysosome, is formed at its tip. This process enables recycling of the lysosomal membrane and maintenance of the lysosome pool necessary for the formation of new autolysosomes, and depends on microtubules and kinesin, the microtubule motor protein [68]. Decreased numbers of lysosomes would result in autophagosome accumulation and a decreased number of autolysosomes, as observed in stressed S6K DKO MEF [30]. Although we cannot rule out such interpretation, it is weakened by the notion that while tubulin acetylation enhances the binding of the microtubule motor proteins, kinesin and dynein, Geeraert et al., Ravikuar et al. and Xu et al. [14,65,67] demonstrated that dynein is the only protein indispensable for an effective autophagosome-lysosome fusion under stress conditions, and that dynein does not participate in ALR. 

In response to the different stress stimuli evaluated in the current study, S6K1 became catalytically inactive, as demonstrated by a reduced phosphorylation of its main substrate, the S6 ribosomal protein (Figure 1). Despite its inactivation, however, S6K1 presence was indispensable for appropriate microtubule acetylation and autophagic flux (current study and [30]). This suggests that its role in tubulin acetylation during stress-induced autophagy is kinase activity-independent. mTORC1-mediated phosphorylation of S6K1 at Thr389 is crucial for the kinase activity [32,69,70]. Interestingly, Rosner et al. [49] showed that phosphorylation at this site is an essential signal targeting S6K1 to the nucleus; by contrast, its dephosphorylation results in the inhibition of the kinase activity and cytoplasmic retention. This is in agreement with our observations that a constitutively active S6K1 variant inhibits autophagy induction in cancer cells [30]. Further, Lee et al., Scott et al. and others [27,71,72,73] demonstrated that activation of a key autophagy component, Ulk1 (Atg1), upon starvation or Atg1 overexpression, blocks Thr389 phosphorylation of S6K1, and that this mechanism is evolutionarily conserved from yeast through *Drosophila* to mammals. These findings support our hypothesis that S6K1 plays a crucial role in tubulin acetylation only under stress conditions, when S6K1 is dephosphorylated and thus, resides in the cytoplasm. Of note, Rosner et al. [49] also demonstrated that the commonly used S6K1 inhibitor, PF-4708671, decouples S6K1 kinase activity from Thr389 phosphorylation, leading to increased S6K1 phosphorylation and, consequently, its translocation to the nucleus, despite the inhibition of its activity. We showed here that such forced phosphorylation of S6K1 (and, probably, its translocation) destroys its kinase-independent function, namely, the role in tubulin acetylation, despite its catalytic inhibition (Figure 9). This emphasizes the notion that the caution must be taken in interpreting the results of experiments involving the PF-4708671 inhibitor, as they might be misleading under certain circumstances. 

**Figure 11:**
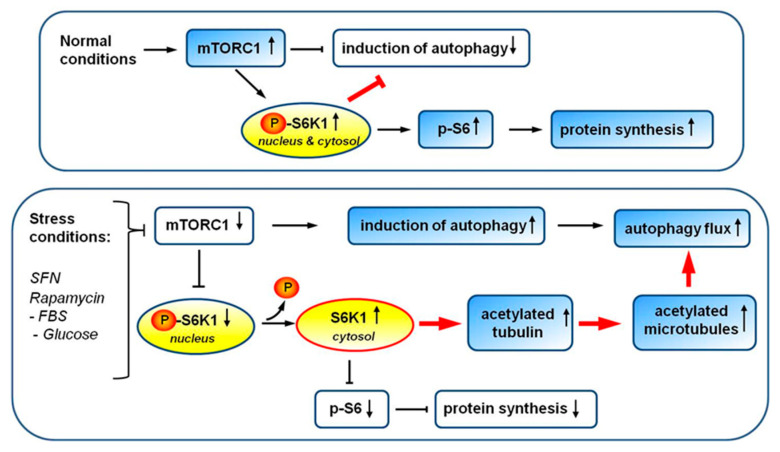
The model of S6K1 role in autophagy under standard (upper panel) and stress (lower panel) conditions. Blue background, activation of a protein/process; white background, inhibition of a protein/process.

In light of the above, we can now paint a more comprehensive picture of the role of S6K1 in autophagy regulation (Figure 11). Specifically, reversible phosphorylation, leading to the change in S6K1 activity and its localization, may act as a switch between the different functions of this protein: (1) promoting translation and inhibiting autophagy when S6K1 is phosphorylated and catalytically active [30]; and (2) upregulating microtubule acetylation and autophagic flux in response to cellular stress when S6K1 is dephosphorylated, kinase-inactive, and localized in the cytoplasm. Such a switch might be additionally regulated by S6K1 acetylation. Indeed, recent studies have reported that such modification of the S6K1 C-terminus attenuates mTORC1-dependent S6K1 phosphorylation [74]. However, it cannot be excluded that S6K1 acetylation contributes to, but is not indispensable for, the kinase-independent protein function(s). This is supported by recent reports demonstrating that, in breast cancer cell lines and tumors overexpressing the splicing factor oncoprotein SRSF1, splicing of mRNA encoding S6K1 is modulated and leads to the expression of short S6K1 isoforms devoid of the majority of the kinase domain and the whole C-terminal domain (which contain the acetylation site). Importantly, even though these short S6K1 variants are kinase-inactive, they possess oncogenic activity, which results from their ability to directly bind mTORC1 and affect its activity [75]. Further, these properties are reiterated by point-substituted full-length kinase-dead S6K1 but not WT protein. Collectively, this indicates that S6K1 plays kinase-independent roles that are facilitated by direct protein binding. Activation or deactivation of S6K1 by a reversible Thr389 phosphorylation appears to be crucial for switching between its different roles. Based on gene disruption analysis and other approaches, Scott et al. [28] proposed that *S6K1* is essential for autophagy in the fat body of *Drosophila*. This appears to contrast with the previous findings in rat hepatocytes that active *S6K1* suppresses autophagy and autophagic flux [25,26]. The kinase-independent functions of S6K1 might explain, at least in part, the discrepancies and conflicting results concerning its role in autophagy obtained using different experimental approaches (e.g., silencing of *S6K1* gene expression or overexpression of a kinase-dead variant), which, in this context, are not equivalent. 

Kinase-independent functions accomplished via protein binding have been demonstrated for such proteins as Ulk1 or hexokinase-II [76,77], in addition to S6K1. In addition to the already described binding of its short and kinase-dead variants to the mTORC1 complex, S6K1 also acts as an actin filament cross-linking protein [78]. Therefore, it cannot be ruled out that S6K1 binds to the microtubular cytoskeleton. This notion is supported by the data of Pavan et al. [79], who showed that S6K1 co-precipitates with tubulin and dynein subunits. Therefore, it is possible that S6K1 plays a structural role, e.g., serving as a platform for the interactions of specific protein(s). Importantly, S6K1-interacting proteins are predominantly related to the “cytoskeleton” and “stress response” [79], which fits well with our hypothesis on the role of S6K in autophagy during stress, mediated by the modulation of microtubule acetylation.

The discovery that S6K1 impacts tubulin acetylation has broad biological implications since both S6K1 and acetylated microtubules are involved in multiple cellular processes, regulating cell growth, metabolism, and development. It has been reported that tubulin acetylation regulates the production and trafficking of matrix metalloproteinase MT1-MMP and stimulates breast tumor cell invasion [80]. Of note, S6K1 is overexpressed, particularly, in breast and ovarian cancers, which correlates with a poor prognosis for the patient. Further, the expression of short, oncogenic, and kinase-inactive S6K1 variants is often observed in breast cancers [75]. Autophagy enables the cell to survive the adverse conditions in solid tumors [81,82], and is also engaged in the processes of cell dedifferentiation and bioenergetic shift from oxidative phosphorylation to anaerobic metabolism [83,84]. This suggests that deregulation of S6K1 might contribute to the development and progression of such cancers, perhaps also via its impact on tubulin acetylation and autophagic flux. Therefore, while the findings of the current study shed new light on the role of S6K1 in cell biology, further studies on the role of this protein in the cell are urgently needed as they can lead to the development of new therapeutic strategies. 

## 5. Conclusions

We showed here that, by controlling tubulin acetylation, S6K1 contributes to the flux of autophagy induced by different stress conditions and in different cells. The presented data strongly suggest that this is a general phenomenon. In addition, this effect appears to be independent of the kinase activity of S6K1. To the best of our knowledge, this is the first report on the impact of S6K1 on tubulin acetylation and microtubular cytoskeleton network.

## Data Availability

The data presented in this study are available on request from the corresponding author.

## Supplementary Materials

The following are available online at https://www.mdpi.com/article/10.3390/cells10040929/s1, Figure S1: Defect in autophagy flux in S6K DKO MEF is not caused by lower levels of autophagy-related proteins; Figure S2: Stress conditions induce stronger accumulation of LC-3 positive vacuoles in cells devoid of S6K than in their WT counterparts.

## Abbreviations

ACF, amino-4-trifluoromethyl coumarin; ALR, autophagic lysosome reformation; ATG1, autophagy-related 1; BSA, bovine serum albumin; ChQ, chloroquine; DMSO, dimethyl sulfoxide; FBS, fetal bovine serum; GFP, green fluorescent protein; HA-S6K1, rapamycin-resistant S6K1 variant fused with hemagglutinin tag; HA, hemagglutinin; HBSS, Hank’s Balanced Salt Solution; HDAC6, histone deacetylase 6; LC3, microtubule-associated proteins 1A/1B light chain 3B; MSK, mitogen and stress-activated protein kinase; MT1-MMP, membrane type 1 matrix metalloproteinase; mTORC1, mammalian/mechanistic target of rapamycin complex 1; PBS, Phosphate Buffered Saline; RSK, 90-kDa ribosomal S6 kinase; S6K DKO MEF, embryonic fibroblasts from S6K1**^−^**^/**−**^ and S6K2**^−^**^/**−**^ double-knockout mouse; S6K1, S6 ribosomal protein kinase 1; S6K2, S6 ribosomal protein kinase 2; S6K, S6K1 and S6K2 kinases; SFN, sulforaphane; Sirt2, NAD-dependent deacetylase sirtuin 2; tubulin K40Q, acetylation-mimicking tubulin variant; tubulin K40R, acetylation-resistant tubulin variant; Ulk1, Unc-51 like autophagy activating kinase; WT MEF, wild-type mouse embryonic fibroblasts; αTAT1, α-tubulin acetyltransferase 1.
