# Supplementary figures and images for: S6K1 Is Indispensible for Stress-Induced Microtubule Acetylation and Autophagic Flux
Source: Cells. 2021 Apr 17;10(4):929. doi: 10.3390/cells10040929 (PMC8073773; doi:10.3390/cells10040929)

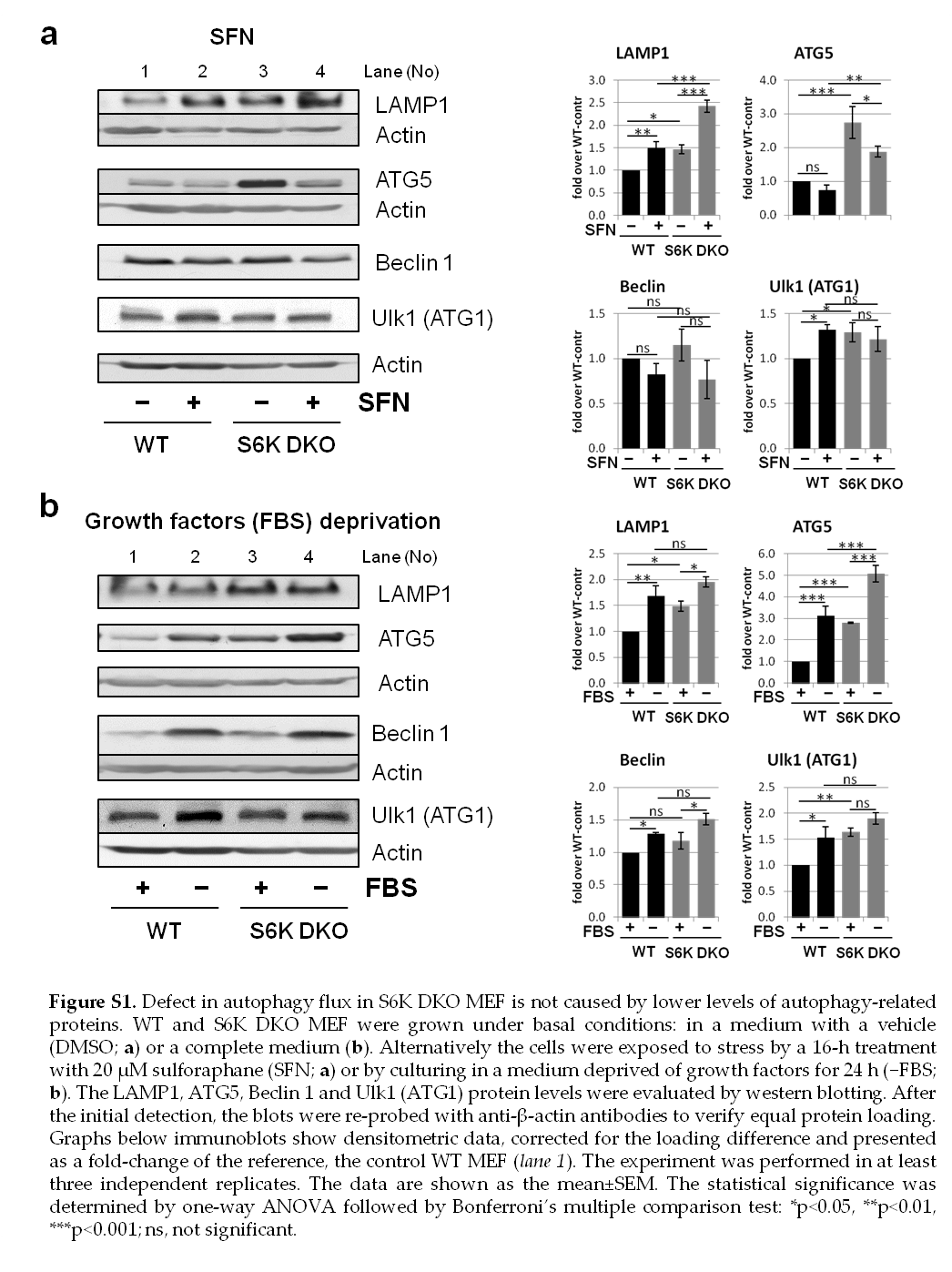

Supplement: Supplementary file 1 [file cells-10-00929-s001.zip › Supplementary Figure S1 cells-1102561 Hac et al.png]

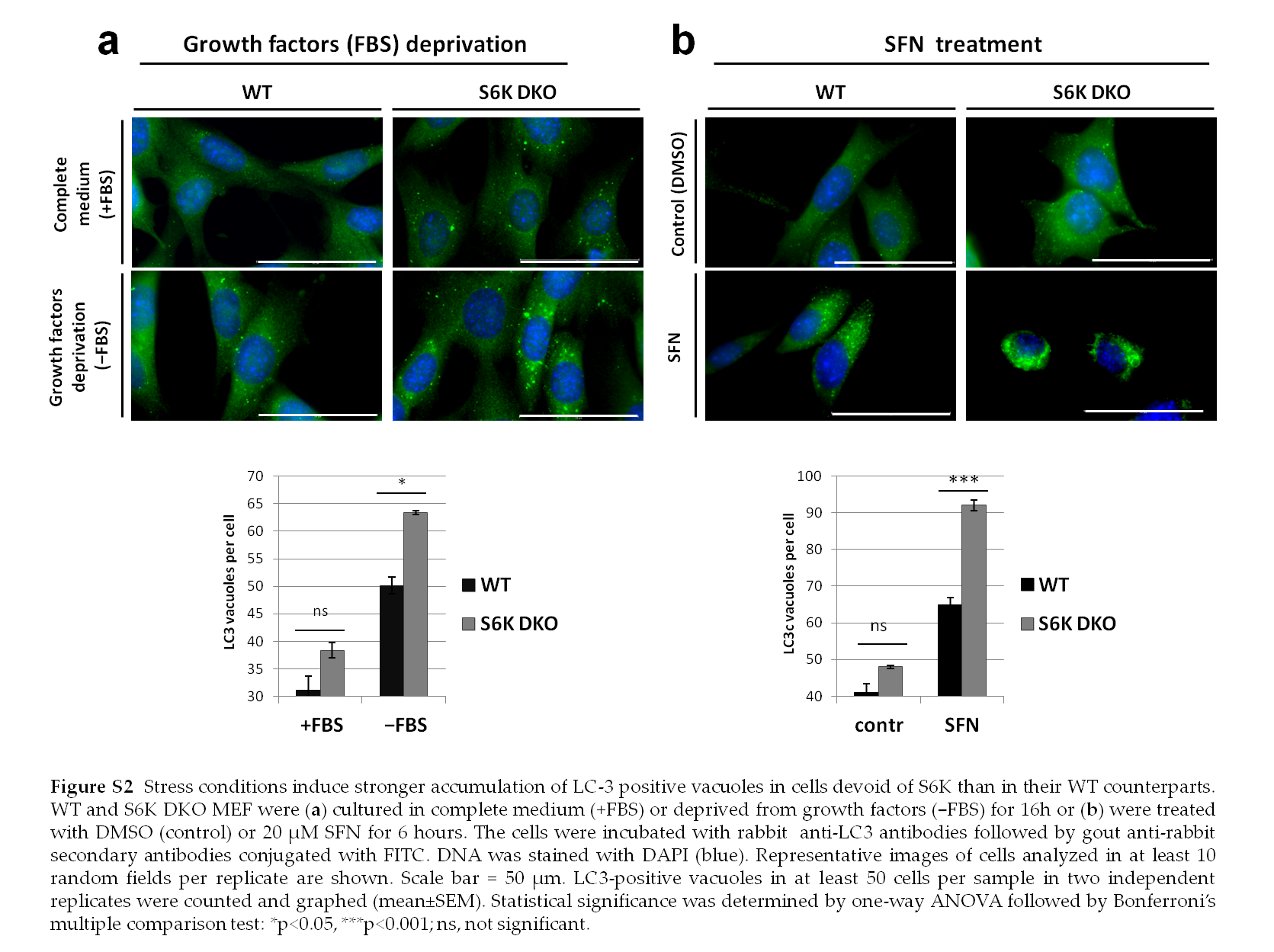

Supplement: Supplementary file 1 [file cells-10-00929-s001.zip › Supplementary Figure S2 cells-1102561 Hac et al.png]
